# Cost-effectiveness analysis of prophylactic laser peripheral iridotomy for primary angle-closure suspect in Japan

**DOI:** 10.1038/s41433-023-02806-3

**Published:** 2023-10-28

**Authors:** Asahi Fujita, Takaaki Konishi, Rei Sakata, Yohei Hashimoto, Hideo Yasunaga, Makoto Aihara

**Affiliations:** 1 Department of Ophthalmology, Graduate School of Medicine, The University of Tokyo, 7-3-1 Hongo, Bunkyo-ku, Tokyo, 113-8655 Japan; 2 Department of Clinical Epidemiology and Health Economics, School of Public Health, The University of Tokyo, Tokyo, Japan

**Keywords:** Quality of life, Optic nerve diseases

## Abstract

**Background/Objectives:**

This study aimed to compare the cost-effectiveness of prophylactic laser peripheral iridotomy (LPI) with that of observation for primary angle-closure suspect (PACS) in Japan.

**Subjects/Methods:**

A Markov model was developed to compare the costs and utilities of prophylactic LPI with those of observation of 40-year-old patients with PACS. In the model with a yearly cycle over a 20-year time horizon, the disease was postulated to irreversibly progress from PACS to primary angle closure, followed by primary angle-closure glaucoma, unilateral blindness, and bilateral blindness. The parameters were estimated mainly based on a recent randomised controlled trial and analyses of Japanese claims data. The incremental cost-effectiveness ratio was estimated from the healthcare payer’s perspective and evaluated at the willingness-to-pay 5 million Japanese Yen per quality-adjusted life-year. The observation period and the age at entry into the cohort was changed to account for a variety of clinical courses in sensitivity analyses. We conducted one-way deterministic sensitivity analysis and probabilistic sensitivity analysis with Monte Carlo simulations with 10 000 iterations.

**Results:**

The incremental cost-effectiveness ratio of LPI was 2,287,662 Japanese Yen (14,298 pounds sterling) per quality-adjusted life-year, which was below the willingness-to-pay threshold. The ratios were approximately 4 and 8 million in the 15-year and 10-year time horizons, respectively. Increasing the age at entry had little influence on the incremental cost-effectiveness ratio. The deterministic and probabilistic sensitivity analyses indicated that the results were robust.

**Conclusions:**

Our results indicate that prophylactic LPI for middle-aged patients with PACS is cost-effective in Japan.

## Introduction

Glaucoma ranks second among the leading causes of blindness worldwide [1]. Among the several types of glaucoma, primary angle-closure glaucoma (PACG) is associated with devastating visual outcomes [2], and 77% of patients with PACG reside in Asia [3]. Primary angle-closure suspect (PACS) represents anatomically narrow angles with no other abnormalities. Some patients with PACS experience an acute angle-closure crisis, whereas a chronic course of PACG development is observed in other patients. Prophylactic laser peripheral iridotomy (LPI) is widely performed in PACS to prevent acute angle-closure crises and the development of PACG in the future. Although the exact prevalence of PACS in Japan is unknown, PACS is reported to be relatively prevalent in Asia (prevalence of 10.4% in China) [4]. Therefore, the cost of universal LPI for PACS is an economic burden in Asia.

According to two recent randomised controlled trials conducted in Asia, prophylactic LPI for PACS decreased the risk of disease progression significantly [5, 6]. However, despite the efficacy of prophylactic LPI, both randomised controlled trials questioned the cost-effectiveness of widespread prophylactic LPI for PACS as the incidence of PACG among patients with PACS was low and recommended that prophylactic LPI should be limited to patients with PACS at high risk of disease progression. Although a previous analysis of the cost-effectiveness of prophylactic LPI in the US demonstrated that prophylactic LPI was cost-effective, the model used a higher disease progression rate than the two recent trials [7]. In addition, no study has investigated the cost-effectiveness of prophylactic LPI in Asia despite the high prevalence of PACS and PACG in Asia. Therefore, the cost-effectiveness of prophylactic LPI should be investigated further, especially in Asian countries.

This study aimed to estimate the cost-effectiveness of prophylactic LPI for PACS using the data from a recent randomised controlled trial conducted in an Asian country on the incidence of PACG and efficacy of LPI [5] and cost in Japan.

## Materials/subjects and methods

### Model overview

This study utilised a health state transition Markov model over a 20-year time horizon from 40 to 59 years of age to evaluate the cost-effectiveness of prophylactic LPI for PACS. We set the minimum age for inclusion in the cohort as 40 years since the prevalence of PACS under the age of 40 was reported to be low [4]. We did not include patients aged ≥60 years as the frequency of cataract surgery increases over the age of 60 [8], and cataract surgery usually improves angle closure without LPI.

**Fig. 1:**
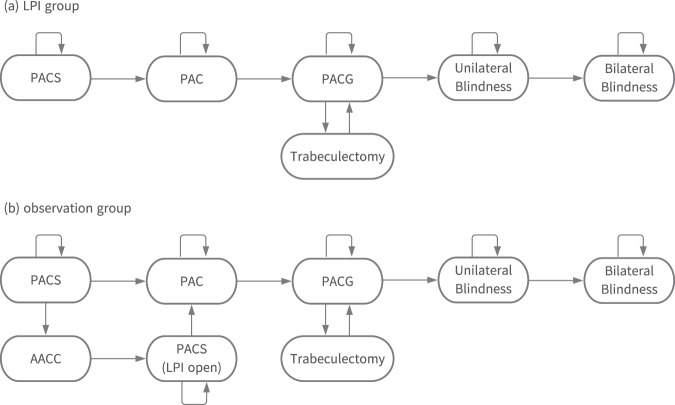
Health states in the Markov model. (**a**) LPI group. (**b**) observation group. The patients in the LPI group (**a**) underwent prophylactic LPI during the first year. The patients in the observation group (**b**) who experienced AACC underwent LPI under emergency settings. LPI laser peripheral iridotomy, PACS primary angle-closure suspect, PAC primary angle-closure, PACG primary angle-closure glaucoma, AACC acute angle-closure crisis.

We developed a yearly cycle Markov model that tracks the transitions of the patients across mutually exclusive health states (Fig. 1). Patients with PACS who underwent prophylactic LPI during the first year were assigned to the LPI group, whereas patients who did not undergo prophylactic LPI were assigned to the observation group. The patients in both groups were at risk of irreversible progression from PACS to primary angle closure (PAC), followed by PACG, unilateral blindness, and bilateral blindness [9–11]. All patients with progression to PAC underwent lens extraction in accordance with the treatment guidelines in Japan [12]. Patients with PACG, whose intraocular pressure could not be controlled with medication, underwent trabeculectomy [13]. In the observation group, patients with PACS were at risk of acute angle-closure crisis and underwent emergency LPI with an intravenous drip of D-mannitol upon the incidence of acute angle-closure crisis [12]. A dead state was not included in the model as the mortality rates for individuals between the ages of 40 and 59 years is very low in Japan [14]. The patients accumulated quality-adjusted life-years (QALYs) and costs during each state, and the transitions occurred according to the input probabilities (Table 1).

**Table 1:** Parameters used in the model.

	Value (range)			Distribution	
	LPI group	Observation group	Data source	Type	SD
Transition probability, %					
PACS to PAC	1.17 (±30%)	2.18 (±30%)	Ref. [5]	Beta	10%
PAC to PACG	0.146 (±30%)	0.34 (±30%)	Ref. [5]	Beta	10%
PACG to Unilateral blindness	0.46 (±30%)		Refs. [9, 13]	Beta	10%
Unilateral blindness to Bilateral blindness	3.87 (±30%)		Refs. [9, 11]	Beta	10%
PACS to AACC	NA	0.097 (±30%)	Ref. [5]	Beta	10%
AACC to PACS (LPI open)	NA	100	NA	NA	NA
PACG to Trabeculectomy (40–49) years old)	0.88 (±30%)		Ref. [15]	Beta	10%
PACG to Trabeculectomy (50–59) years old)	0.63 (±30%)		Ref. [15]	Beta	10%
Utility, QALY					
PACS	1		Ref. [9]	NA	NA
AACC	0.99		Ref. [18] and speculation	NA	NA
PAC	1		Ref. [9]	NA	NA
PACG	0.75 (±8%)		Ref. [18]	Beta	5%
Trabeculectomy	0.74 (±8%)		Ref. [20]	Beta	5%
Unilateral blindness	0.47 (±8%)		Ref. [19]	Beta	5%
Bilateral blindness	0.26 (±8%)		Ref. [19]	Beta	5%
Cost, JPY					
PACS (LPI, 1st year)	98 393 (±20%)		Ref. [12]	Gamma	10%
PACS (observation, 1st year)	19 560 (±20%)		Ref. [12]	Gamma	10%
PACS (LPI, observation (LPI open), subsequent years)	23 780 (±20%)		Ref. [12]	Gamma	10%
PACS (observation, subsequent years)	22 180 (±20%)		Ref. [12]	Gamma	10%
PAC (1st year)	171 259 (±20%)		Ref. [12]	Gamma	10%
PAC (subsequent years)	23 780 (±20%)		Ref. [12]	Gamma	10%
PACG (40–49 years)	48 545 (±20%)		Ref. [12]	Gamma	10%
PACG (50–59 years)	47 553 (±20%)		Ref. [12]	Gamma	10%
Unilateral blindness (40–49 years) old)	46 645 (±20%)		Ref. [12]	Gamma	10%
Unilateral blindness (50–59 years) old)	45 653 (±20%)		Ref. [12]	Gamma	10%
Bilateral blindness (40–49 years)	44 745 (±20%)		Ref. [12]	Gamma	10%
Bilateral blindness (50–59 years)	43 753 (±20%)		Ref. [12]	Gamma	10%
AACC	107 167 (±20%)		Ref. [12]	Gamma	10%
Trabeculectomy	697 008 (±20%)		Ref. [17]	Gamma	10%
Discount rate, %	2.0 (0.0–4.0)		Ref. [16]	NA	NA

*LPI* laser peripheral iridotomy, *PACS* primary angle-closure suspect, *PAC* primary angle closure, *PACG* primary angle-closure glaucoma, *AACC* acute angle-closure crisis, *JPY* Japanese Yen, *QALY* quality-adjusted life-year, *NA* not applicable.

### Input parameters

The transition probabilities of PACS to PAC and PACG for each group were obtained from a recent randomised controlled trial [5]. The data regarding the progression rate from PACG to unilateral and bilateral blindness were obtained from a previous study that performed a cost-effectiveness analysis of population-based glaucoma screening in China [10]. The transition probability of PACG to trabeculectomy was obtained from a previous study that analysed the Japanese claims database [15].

All costs were recorded from the healthcare payer’s perspective in Japanese Yen (JPY), in accordance with the Japanese guideline for evaluation of cost-effectiveness [16]. The costs were based on the medical fees provided by the government as of May 2022. Supplementary Table 1 presents the procedure codes and costs included in the calculations. The annual cost of the drugs used in the treatment of PACG was obtained from a cost analysis study conducted in Japan [15]. The costs of trabeculectomy and 1-year postoperative treatment were obtained from another cost analysis study conducted in Japan [17]. The one-year treatment cost for each health state was inferred from clinical practice based on the guidelines for glaucoma treatment in Japan (Supplementary Table 2) [12].

The utility values for each health state were obtained from previous studies [9, 18, 19]. A utility loss of 0.007 was applied in the case of glaucoma surgery [20].

### Analysis

The incremental cost-effectiveness ratio (ICER) was calculated as the incremental cost per QALY of prophylactic LPI from the healthcare payer’s perspective using a Markov model. ICER was evaluated at a commonly used willingness-to-pay threshold in Japan (5 million JPY per QALY) [21]. The exchange rate as of 20 January 2023 was used in this study (160 JPY = 1 pound sterling, £). An annual discount rate of 2% was applied to the costs and outcomes [16]. All analyses were conducted using TreeAge Pro 2023 software (TreeAge Software, Williamstown, MA, USA).

Six scenarios with different ages at entry and exit from the cohort (40–59 years in the base-case analysis) were used for the sensitivity analysis: 40–54 years (15-year horizon), 40–49 years (10-year horizon), 50–69 years (20-year horizon), 50–64 years (15-year horizon), 50–59 years (10-year horizon), and 40–99 years (60-year horizon). The age at entry was changed to simulate cases in which PACS was detected at the age of 50 years. The age at exit was changed to account for the variations in the timing of cataract surgery in the first five scenarios and to estimate the lifetime cost-effectiveness in the last scenario.

A one-way deterministic sensitivity analysis was performed to determine the impact of transition probabilities, utilities, and costs. A deviation of 30%, 8%, and 20% was assigned for transition probabilities, utilities, and cost, respectively [9, 22]. A discount rate of 0–4% was used based on the Japanese guidelines [16]. A tornado diagram was created to show the 12 factors to which ICER was sensitive. Considering the higher transition probabilities of PACS to PAC and PAC to PACG applied in a previous cost-effectiveness study [7], an additional deterministic sensitivity analysis was performed with the probabilities applied in that study.

Probabilistic sensitivity analysis was performed using Monte Carlo simulations with 10,000 iterations. Beta distributions with standardised differences of 10% and 5% were adopted for transition probabilities and utilities, respectively [22]. Gamma distributions with a standardised difference of 10% were adopted for costs. Acceptability curves depicting the probability of prophylactic LPI being cost-effective based on the basis of willingness-to-pay were created.

Lastly, an analysis was performed from a societal perspective. Disability pension, care cost, in-kind benefit, community care, and average salary based on a previous cost-effectiveness analysis regarding blindness in Japan were included as indirect costs [23]. In addition, a travel cost of 4547 JPY per visit [24] was also included in the sensitivity analysis. It was assumed that unilateral blindness incurred 30% of the indirect costs of bilateral blindness [9].

### Ethics

This analysis was performed in accordance with the Consolidated Health Economic Evaluation Reporting Standards and the relevant Japanese guidelines [16, 25].

## Results

Table 2 presents the results of the base-case and the six scenarios with different ages at entry and exit from the cohort. The ICER was 2,287,662 JPY (£14,298) per QALY in the base-case analysis, which is below the willingness-to-pay threshold (5 million JPY per QALY). In contrast, the ICER was approximately 7,600,000 JPY (£47,500) per QALY and above the willingness-to-pay threshold under the 10-year time horizon. The ICER showed no significant changes on increasing the age at entry into the cohort to 50 years of age. The ICER was 124,675 JPY (£779) per QALY under the 60-year time horizon, which was lower than the willingness-to-pay threshold.

**Table 2:** Cost-effectiveness of prophylactic LPI versus that of observation (base-case and scenarios with different ages at entry and exit from the cohort).

Observation period	Strategy	Cost (JPY)	Incremental cost (JPY)	QALY	Incremental QALY	ICER (JPY/QALY)
40–59 years of age (20-year horizon, base-case)	LPI	449,050	55,572	16.67	0.02	2,287,662
	Observation	393,478		16.65		
40–54 years of age (15-year horizon)	LPI	368,602	60,784	13.10	0.02	3,958,450
	Observation	307,818		13.09		
40–49 years of age (10-year horizon)	LPI	280,195	66,526	9.16	0.01	7,610,359
	Observation	213,669		9.15		
50–69 years of age (20-year horizon)	LPI	449,037	55,615	16.67	0.02	2,294,626
	Observation	393,421		16.65		
50–64 years of age (15-year horizon)	LPI	368,596	60,803	13.10	0.02	3,966,008
	Observation	307,794		13.09		
50–59 years of age (10-year horizon)	LPI	280,194	66,531	9.16	0.01	7,617,443
	Observation	213,663		9.15		
40–99 years of age	LPI	880,510	23,196	35.40	0.19	124,675
(60-year horizon)	Observation	857,314		35.21		

*LPI* laser peripheral iridotomy, *JPY* Japanese Yen, *QALY* quality-adjusted life-year, *ICER* incremental cost-effectiveness ratio.

**Fig. 2:**
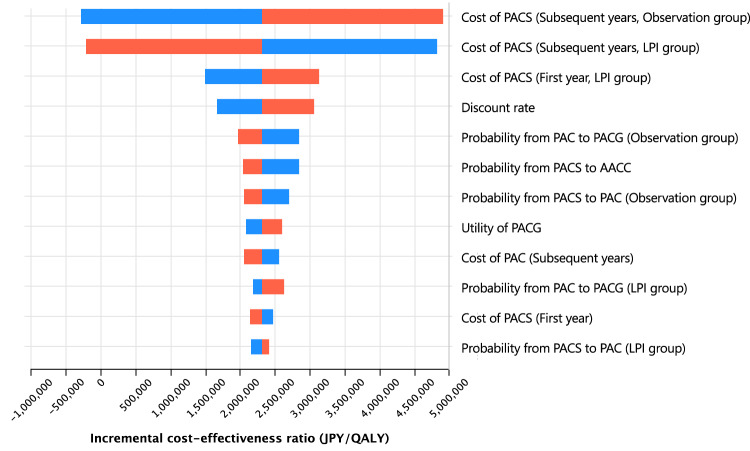
Tornado diagram for the incremental cost-effectiveness ratio of prophylactic LPI compared with that of observation. The red bar shows the variation on increasing the parameter. The blue bar shows the variation on decreasing the parameter. The upper parameters had a greater impact on the incremental cost-effectiveness ratio. LPI laser peripheral iridotomy, PACS primary angle-closure suspect, PAC primary angle-closure, PACG primary angle-closure glaucoma, AACC acute angle-closure crisis.

Figure 2 presents the results of the one-way deterministic sensitivity analysis as a tornado diagram. The costs of PACS follow-up had the greatest influence on ICER. However, no parameter could increase the ICER above a threshold of 5 million JPY per QALY. The ICER in the additional deterministic sensitivity analysis, in which higher transition probabilities from a previous study were applied, was lower than that in the base-case analysis (Supplementary Table 3).

**Fig. 3:**
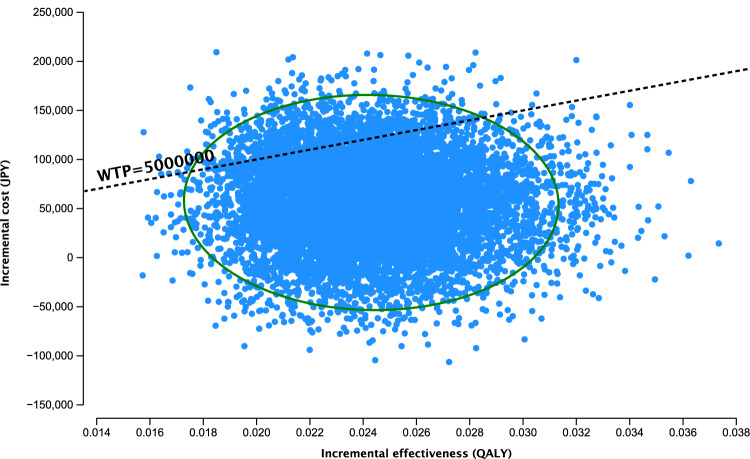
Scatter plot based on probabilistic sensitivity analysis for the cost-effectiveness of prophylactic LPI compared with that of observation. LPI laser peripheral iridotomy, JPY Japanese Yen, QALY quality-adjusted life-year.

In the probabilistic sensitivity analysis, 89.4% of the ICER estimates were below the willingness-to-pay threshold of 5 million JPY per QALY, and approximately 10% were located in the dominant area (Fig. 3). Supplementary Fig. 1 presents the acceptability curve showing the probability of being cost-effective according to the willingness-to-pay threshold. Prophylactic LPI was more likely to be cost-effective than observation when the willingness-to-pay threshold exceeded approximately 2.3 million JPY (approximately £14,375).

The ICER was 2,134,471 JPY (£13,340) per QALY according to the analysis conducted from the societal perspective.

## Discussion

This study investigated the cost-effectiveness of prophylactic LPI for PACS versus that of observation using a Markov model. The ICER was 2,287,662 JPY (£14,298) per QALY, indicating that prophylactic LPI was cost-effective. These results were confirmed to be robust by several sensitivity analyses.

The prevalence of PACG in Asia is estimated to be 1.09%, which is more than double the global prevalence, indicating that Asia has the highest prevalence of PACG [3]. In addition, Asia accounts for approximately 60% of the world’s total population [26]. Therefore, it is important to assess the cost-effectiveness of prophylactic treatment for PACG in Asia. Two randomised controlled trials conducted in China and Singapore reported that prophylactic LPI for PACS was effective in preventing disease progression [5, 6]. Nevertheless, the cost-effectiveness of widespread prophylactic LPI has been questioned since the incidence of PAC and PACG was very rare. Our cost-effectiveness analysis indicates that prophylactic LPI for middle-aged patients with PACS is likely to be cost-effective in Japan, and as the incidence of adverse events after LPI was reportedly very low [5, 6], prophylactic LPI for middle-aged patients with PACS can be recommended.

Cataracts are a trigger for narrow angles, and cataract surgery can improve angle closure [27] and reduce the intraocular pressure; [28] however, some patients experience glaucomatous progression of PACS after cataract surgery [29]. The influence of cataract and cataract surgery on the disease progression remains unclear; therefore, the cost-effectiveness of prophylactic LPI was evaluated in a population that was unlikely to be affected by cataracts, that is, a population aged 40–59 years. Thus, the main analysis demonstrates the cost-effectiveness of LPI irrespective of cataract. Moreover, the scenarios with different ages at entry demonstrated that ICERs under the 10-year horizon exceeded the willingness-to-pay threshold, whereas those under the 15- and 20-year horizons were below the threshold. These results indicate that prophylactic LPI is unlikely to be cost-effective for eyes with progressive cataracts that would require cataract surgery within 15 years.

Since glaucoma is a chronic disease, the lifetime cost-effectiveness plays an important role in treatment selection. It was estimated that the lifetime cost-effectiveness of prophylactic LPI would be significantly lower than the willingness-to-pay threshold (124,675 JPY per QALY under the 60-year time horizon), although the impact of cataract surgery on the disease progression was not incorporated owing to the lack of data. Cataract surgery would impact the transition probabilities by slowing the disease progression. As the one-way sensitivity analysis indicated that the changes in transition probabilities would have a small effect on ICER, it was speculated that prophylactic LPI would remain cost-effective in the lifetime horizon even if the impact of cataract surgery was considered.

Lens extraction has been recommended as a treatment for PACG based on its efficacy and cost-effectiveness compared with therapeutic LPI [30]. Some ophthalmologists suggest that lens extraction is also more effective than LPI as a prophylactic procedure for PACS; however, there has been little evidence to support this assumption [12], thereby warranting further analyses comparing the efficacy and cost-effectiveness of prophylactic LPI and lens extraction for PACS.

A cost-effectiveness analysis of prophylactic LPI in the US reported that the ICER of LPI versus that of observation was $2,915,165 per QALY under the 2-year horizon and that LPI overshadowed observation after 3 years up to the age of 50 years. Our results provided less cost-effective estimates of LPI than theirs. Two possible reasons could explain this discrepancy. First, the data source for the transition probability of PACS to PAC was different. The previous study used a transition probability based on a previous cost-effectiveness model of glaucoma screening [9], which referred to three observational studies published in 1992 and 2003 [31–33]. In contrast, the present study used the progression rate reported in a randomised controlled trial that was published in 2022 [5]. As a result, the transition probabilities that were applied in our model were less than half of those applied in their study. However, the main findings of the sensitivity analysis in the present study did not vary on using the transition probabilities used in their study. Second, the cost parameters were generally lower in our study compared with those in their study, possibly due to the differences between the two countries in terms of the status of medical care and the different perspectives used in the analysis (healthcare payer’s perspective in our study versus societal perspective in their study). As the cost parameters in the current study were based on our previous study using claims databases in Japan, we believe that our analysis accurately reflected the real-world clinical practice. Although the one-way sensitivity analysis revealed that the costs for the PACS follow-ups had the greatest influence on ICER, the ICER did not exceed the willingness-to-pay threshold in the worst situations. The ICER estimated from a societal perspective was also lower than the willingness-to-pay threshold in the present study, indicating that prophylactic LPI would be cost-effective both from the societal and healthcare payer’s perspectives.

There are several mechanisms underlying narrow angles, such as relative pupillary block, lens thickness and position, and plateau iris. Prophylactic LPI is effective in cases with relative pupillary block; however, the efficacy of LPI in cases with plateau iris is limited. A previous study demonstrated the persistence of plateau iris after peripheral iridotomy in 26% of preoperative plateau iris configurations [34]. Since the data source for the efficacy of prophylactic LPI in our analysis did not distinguish between narrow-angle mechanisms, the analysis in the present study estimated the cost-effectiveness of LPI for PACS as a whole. The efficacy of prophylactic LPI for PACS of each mechanism should be clarified in the future to improve treatment strategies.

The results of the present study regarding the cost-effectiveness of prophylactic LPI can be generalised to some countries. For example, in Korea, the cost of glaucoma treatment is comparable with that in Japan, and the willingness-to-pay threshold is estimated to be approximately 3 million JPY (30,000,000 South Korean Won) per QALY [35]. Consequently, prophylactic LPI for PACS in Korea would be cost-effective as the probability of LPI being cost-effective was approximately 65% at the Korean threshold. Several costs in China were beyond the ranges in the current analysis; for example, the estimated treatment costs of PACS, PAC, and PACG were lower, whereas those of unilateral and bilateral blindness were higher than their counterparts in Japan [9]. The one-way deterministic sensitivity analysis showed that the costs of blindness hardly influenced the ICER; in contrast, the decreased first-year cost of PACS in the LPI group reduced the ICER. Therefore, as the willingness-to-pay threshold in China was estimated as 23,850 US dollars (approximately 3 million JPY) per QALY [9], prophylactic LPI would also be cost-effective.

Our study had some limitations. First, although we attempted to estimate the parameters using real-world data in Japan, we were unable to obtain some parameters, such as utilities and the transition probabilities. We referred to studies conducted in Asian countries in such situations. Second, we were unable to consider the influence of cataracts and cataract surgery. Cataract surgery can be performed before the age of 60 years in some cases showing early progress. The cost-effectiveness of prophylactic LPI in such patients remains unknown. Third, the timings of the transitions are not necessarily at the end of each cycle. However, half-cycle correction was not performed as the correction reportedly changes the ICER by <1% [36]. Lastly, although the results are possibly generalisable to Korean and Chinese populations, they cannot be generalised to populations across the world.

In conclusion, compared with observation, prophylactic LPI for middle-aged patients with PACS was estimated to be cost-effective in Japan.

## Summary

### What was known before

- Prophylactic laser peripheral iridotomy for primary angle-closure suspect was effective in reducing the progression to primary angle closure and primary angle-closure glaucoma.
- Although the prevalence of primary angle-closure suspect is high in Asia, the cost-effectiveness of prophylactic laser peripheral iridotomy for primary angle-closure suspect remains unknown.

### What this study adds

- Our results indicate that prophylactic laser peripheral iridotomy for middle-aged patients with primary angle-closure suspects is cost-effective in Japan.
- Prophylactic laser peripheral iridotomy can be recommended for middle-aged patients with primary angle-closure suspect.

### Supplementary information

Supplementary material
