# Supplementary material for: Cost-effectiveness analysis of prophylactic laser peripheral iridotomy for primary angle-closure suspect in Japan
Source: Eye (Lond). 2023 Oct 28;38(5):930–6. doi: 10.1038/s41433-023-02806-3 (PMC10966023; doi:10.1038/s41433-023-02806-3)
Supplement: Supplementary file 1 — Supplementary material [file 41433_2023_2806_MOESM1_ESM.docx]

**Supplementary Table 1. Costs of procedures, examinations, and office visits**

|  | Procedure codes | Costs  (Japanese Yen) |
| --- | --- | --- |
| **Procedures** |  |  |
| Trabeculectomy | 150335910 | 236 000 |
| Lens extraction | 150253010 | 121 000 |
| **Examinations** |  |  |
| Visual field testing (static, unilateral) | 160082210 | 2 900 |
| Visual field testing (dynamic, unilateral) | 160082010 | 1 950 |
| Visual field testing (dynamic, bilateral) | 160082130 | 3 900 |
| Slit-lamp examination (anterior chamber) | 160084510 | 480 |
|  | 160084650 |  |
|  | 160146550 |  |
| Slit-lamp examination (anterior and posterior chamber) | 160081610 | 1 120 |
| Visual acuity testing | 160082810 | 690 |
|  | 160179110 |  |
| Intraocular pressure measurement | 160082910 | 820 |
| Funduscopy (unilateral) | 160081010 | 560 |
| Funduscopy (bilateral) | 160081130 | 1 120 |
| Fundus photography | 160203810 | 580 |
| Optical coherence tomography | 160183310 | 2 000 |
| Anterior segment optical coherence tomography | 160213610 | 2 650 |
| Gonioscopy (without pressure) | 160084710 | 380 |
| Gonioscopy (with pressure) | 160084810 | 760 |
| Specular microscopy | 160148310 | 1 600 |
| Axial length measurement | 160187310 | 1 500 |
| **Office visits** |  |  |
| First office visit | 111000110 | 2 880 |
| Follow-up office visit | 112007410 | 730 |

The costs are based on medical fees as of May 2022. In cases where multiple costs could be applied to one procedure/examination/office visit, the cost expected to be most frequent was selected as the representative.

**Supplementary Table 2. Breakdown of the one-year treatment cost for each health state inferred from the guidelines**

| Health State | Medical Service | Annual Frequency |
| --- | --- | --- |
| PACS (LPI, 1st year) | New office visit^1^ | 1 |
|  | LPI^4^ | 1 |
|  | Office visit (without pupil dilation)^2^ | 1 |
| PACS (observation, 1st year) | New office visit^1^ | 1 |
|  | Office visit (without pupil dilation)^2^ | 1 |
| PACS (LPI, observation (LPI open), subsequent years) | Office visit (without pupil dilation)^2^ | 1 |
|  | Office visit (with pupil dilation)^3^ | 1 |
|  | Visual field testing (static, unilateral) | 2 |
|  | Specular microscopy | 1 |
|  | Anterior segment optical coherence tomography | 2 |
|  | Optical coherence tomography | 1 |
|  | Gonioscopy (without pressure) | 2 |
| PACS (observation, subsequent years) | Office visit (without pupil dilation)^2^ | 1 |
|  | Office visit (with pupil dilation)^3^ | 1 |
|  | Visual field testing (static, unilateral) | 2 |
|  | Anterior segment optical coherence tomography | 2 |
|  | Optical coherence tomography | 1 |
|  | Gonioscopy (without pressure) | 2 |
| PAC (1st year) | Lens extraction^5^ | 1 |
|  | Office visit (without pupil dilation)^2^ | 1 |
|  | Office visit (with pupil dilation)^3^ | 1 |
|  | Visual field testing (static, unilateral) | 2 |
|  | Specular microscopy | 2 |
|  | Anterior segment optical coherence tomography | 2 |
|  | Optical coherence tomography | 1 |
|  | Gonioscopy (without pressure) | 2 |
| PAC (subsequent years) | Office visit (without pupil dilation)^2^ | 1 |
|  | Office visit (with pupil dilation)^3^ | 1 |
|  | Visual field testing (static, unilateral) | 2 |
|  | Specular microscopy | 1 |
|  | Anterior segment optical coherence tomography | 2 |
|  | Optical coherence tomography | 1 |
|  | Gonioscopy (without pressure) | 2 |
| PACG | Office visit (without pupil dilation)^2^ | 3 |
|  | Office visit (with pupil dilation)^3^ | 1 |
|  | Visual field testing (static, unilateral) | 4 |
|  | Specular microscopy | 1 |
|  | Anterior segment optical coherence tomography | 2 |
|  | Optical coherence tomography | 1 |
|  | Gonioscopy (without pressure) | 2 |
|  | Medication^6^ | 1 |
| Unilateral blindness | Office visit (without pupil dilation)^2^ | 3 |
|  | Office visit (with pupil dilation)^3^ | 1 |
|  | Visual field testing (static, unilateral) | 2 |
|  | Visual field testing (dynamic, unilateral) | 2 |
|  | Specular microscopy | 1 |
|  | Anterior segment optical coherence tomography | 2 |
|  | Optical coherence tomography | 1 |
|  | Gonioscopy (without pressure) | 2 |
|  | Medication^6^ | 1 |
| Bilateral blindness | Office visit (without pupil dilation)^2^ | 3 |
|  | Office visit (with pupil dilation)^3^ | 1 |
|  | Visual field testing (dynamic, bilateral) | 2 |
|  | Specular microscopy | 1 |
|  | Anterior segment optical coherence tomography | 2 |
|  | Optical coherence tomography | 1 |
|  | Gonioscopy (without pressure) | 2 |
|  | Medication^6^ | 1 |
| AACC | LPI^4^ | 1 |
|  | Office visit (without pupil dilation)^2^ | 1 |
|  | Office visit (with pupil dilation)^3^ | 1 |
|  | D-Mannitol 75g | 1 |
|  | Acetazolamide Sodium 500mg | 14 |

^1^ The cost of new office visits included the costs of the first office visit, visual acuity testing, intraocular pressure measurement, slit-lamp examination (anterior and posterior chamber), bilateral funduscopy, gonioscopy (with pressure), specular microscopy, axial length measurement, anterior segment optical coherence tomography, fundus photography, and optical coherence tomography.

^2^ The cost of office visits without dilation included the costs of follow-up office visits, visual acuity testing, intraocular pressure measurement, slit-lamp examination (anterior chamber), and bilateral fundoscopy.

^3^ The cost of office visits with dilation included the costs of follow-up office visits, visual acuity testing, intraocular pressure measurement, slit-lamp examination (anterior and posterior chamber), and bilateral fundoscopy.

^4^ The cost of LPI included the costs of the LPI procedure, three office visits without dilation, one bottle of 2% pilocarpine hydrochloride, one bottle of apraclonidine hydrochloride, and one bottle of 1% betamethasone sodium phosphate.

^5^ The cost of lens extraction included the costs of the lens extraction procedure, two office visits with dilation, two office visits without dilation, two sessions of specular microscopy, axial length measurement, two sessions of optical coherence tomography, two bottles of 1% betamethasone sodium phosphate, two bottles of moxifloxacin hydrochloride, and two bottles of nepafenac.

^6^ The cost of medication was obtained from a previous study that performed cost analysis of the Japanese claims database. (Fujita A, Hashimoto Y, Matsui H, et al. Recent trends in treatment and associated costs of primary angle-closure glaucoma: A retrospective cohort study. *Ophthalmol Glaucoma.* 2023 May-Jun;6(3):308-315.)

Abbreviations: LPI, laser peripheral iridotomy; PACS, primary angle-closure suspect; PAC, primary angle closure; PACG, primary angle-closure glaucoma; AACC, acute angle-closure crisis.

**Supplementary Table 3. Results of sensitivity analysis adapting higher transition probabilities in a previous study**

| Strategy | Cost (JPY) | Incremental  cost (JPY) | QALY | Incremental  QALY | ICER  (JPY/QALY) |
| --- | --- | --- | --- | --- | --- |
| LPI | 461 106 | 52 276 | 16.57 | 0.09 | 595 504 |
| Observation | 408 829 |  | 16.48 |  |  |

Abbreviations: LPI, laser peripheral iridotomy; JPY, Japanese Yen; QALY, quality-adjusted life-year; ICER, incremental cost-effectiveness ratio.

**Supplementary Figure 1. Acceptability curve based on the probabilistic sensitivity analysis for the cost-effectiveness of prophylactic LPI compared with that of observation**

Abbreviations: LPI, laser peripheral iridotomy; JPY, Japanese Yen
